# DMM Outstanding Paper Prize 2022 winners: Tamihiro Kamata, Jennifer K. Sargent and Mark A. Warner

**DOI:** 10.1242/dmm.050268

**Published:** 2023-05-05

**Authors:** Rachel Hackett

**Affiliations:** The Company of Biologists, Bidder Building, Station Road, Cambridge CB24 9LF, UK

## Abstract

Disease Models & Mechanisms (DMM) is delighted to announce that the winners of the DMM Outstanding Paper Prize 2022 are Tamihiro Kamata for their Research Article (titled ‘ Statins mediate anti- and pro-tumourigenic functions by remodelling the tumour microenvironment’), and Jennifer K. Sargent and Mark A. Warner for their Resource Article (titled ‘ Genetically diverse mouse platform to xenograft cancer cells’). The two prizes of £1000 are awarded to the first author(s) of the papers that are judged by the journal's Editors to be the most outstanding contribution to the journal that year.

## Teaser

**Summary:** DMM announces the winners of the DMM Outstanding Paper Prize for Research and Resource articles published in 2022.

## Outstanding Paper Prize winner for Research Articles: Tamihiro Kamata

Tamihiro is a Japanese physician-scientist specializing in haematology/oncology. He obtained his MD at Keio University School of Medicine in Tokyo, Japan. He then worked as a clinician for nine years in Japan, where he treated patients with haematological malignancies using intensive chemotherapy, including allogeneic/autologous stem cell transplantation. During this time, he also gained valuable experience in basic science by conducting research on retroviral vectors for haematopoietic stem cell gene therapy (Kamata et al., 2002).

**Figure:**
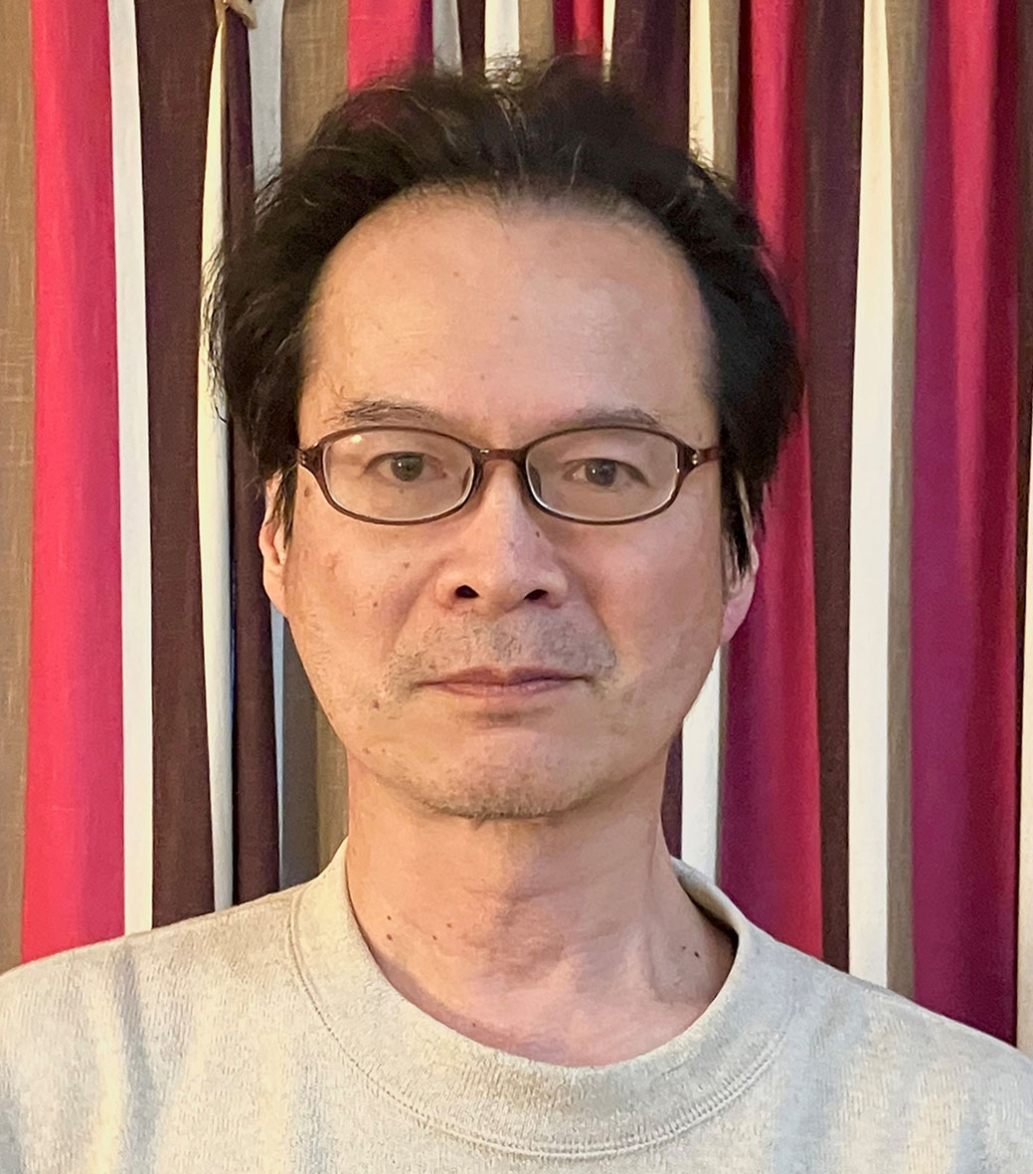
Tamihiro Kamata

In 2001, Tamihiro moved to the USA to work as a research scientist at the University of California, San Francisco (UCSF), under the supervision of Prof. Andrew Leavitt. During his time at UCSF, he focused on investigating the role of RAF kinases (CRAF and BRAF) in haematopoiesis using various genetic approaches, such as knockout mouse models and *in vitro* differentiation systems for gene-targeted embryonic stem cells (Kamata et al., 2004, 2005). Following the discovery of BRAF mutations in human cancers in 2002, Tamihiro and colleagues expanded their transgenic animal studies to include BRAF^V600E^-driven cancer models, in collaboration with Prof. Martin McMahon (Kamata et al., 2013).

In 2006, Tamihiro relocated to the UK to join Prof. Catrin Pritchard's group at the University of Leicester, where his research focused on oncogene-driven cancer models. After Tamihiro's initial four years at the University of Leicester investigating tumourigenic functions of the kinase-dead (class III) BRAF mutation (Kamata et al., 2010), Tamihiro and colleagues turned their attention towards the characterization of the alveolar macrophage (AM)-like tumour-associated macrophages (TAMs) observed in their BRAF^V600E^-driven early lung tumour model (Kamata et al., 2015). Notably, their discovery of the interactive secretome between tumour cells and AM-like TAMs in this model, through cholesterol-regulating Niemann–Pick C2 protein and CC chemokine receptor 1 (CCR1) ligands, directed their current study investigating pro- and anti-tumourigenic effects of statins (Kamata et al., 2022). They found that the chemo-preventive effects of statins achieved by targeting CCR1-dependent AM-like TAMs could be cancelled when continuously dosed in advanced cancers, leading to TAM replacement with monocyte-derived populations and accompanied by robust re-organization of the tumour immune microenvironment (Kamata et al., 2022).

Tamihiro's future research will focus on the identification and characterization of AM-like TAMs in advanced human lung adenocarcinoma, including pre-clinical evaluation of co-targeting of AM-like TAMs (with statins/CCR1 inhibitors) and the tumour immune microenvironment (with immune checkpoint blockers) for human lung adenocarcinoma, using a patient-derived explant culture platform.

## Outstanding Paper Prize winners for Resource Articles: Jennifer K. Sargent and Mark A. Warner

### Jennifer K. Sargent

Jennifer started her biology career in high school when she worked with the Community Environmental Health Lab (CEHL) located at the Mount Desert Island Biological Laboratory (MDIBL) in Bar Harbor, Maine, USA. While working at the MDIBL, she assisted in teaching second and third graders with Dr Jane Disney about water-quality monitoring. She worked with the town of Bar Harbor conducting swim-beach surveys monitoring the levels of coliforms in the area and provided data to locals and tourists about the findings. Later in the internship with MDIBL, she began red-tide monitoring by netting phytoplankton and submitting her results to the State of Maine.

**Figure:**
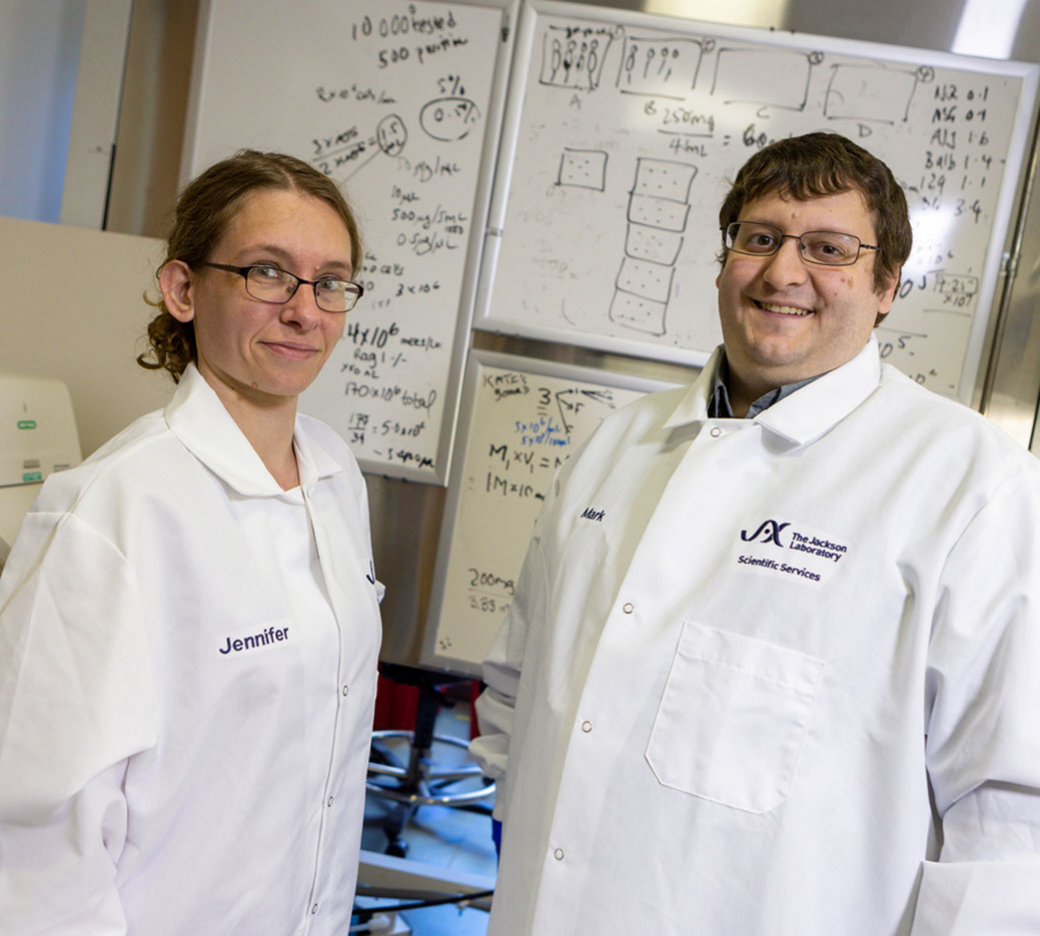
Jennifer K. Sargent and Mark A. Warner

Directly after high school, Jennifer went to the University of Maine at Machias, where she studied marine biology – specifically mariculture. She interned at the Downeast Institute (DEI) on Great Wass Island doing hatchery maintenance. Following her time at DEI, she volunteered to conduct research on pH levels from acid rain and its effects on Atlantic salmon gills at the Downeast Salmon Federation (DSF). She graduated with a bachelor's degree in marine biology with a concentration in mariculture.

After working with the DSF, Jennifer worked at the Cooperative Center for Aquaculture Research (CCAR) in Franklin, Maine. Next, she worked for the State of Maine conducting red-tide monitoring for the Department of Marine Resources (DMR) in her hometown of Lamoine, Maine. There, they used mice as a bioassay to determine the toxicity levels of clams and mussels that were contaminated with red-tide organisms.

Jennifer moved on to working with mice at The Jackson Laboratory (JAX) in Bar Harbor. She started her career at JAX in the mouse rooms, making up breeding schemes, normal mouse husbandry tasks and shipping mice to external customers. Her husbandry experience motivated her to study cancer aetiology. Jennifer was hired as a xenograft technician within the Patient-Derived Xenograft Research and Development Core (PDX R&D Core). She now uses this opportunity to make a difference in the cancer research field. In the PDX R&D Core, she continues to learn many surgical techniques and conducts the testing of compounds against different cancer types. She contributed to a major study that tested a new chemotherapy for leukaemia, which is now in US clinical trials (NCT03997968).

When working with these compounds in NOD.Cg-*Prkdc^scid^ Il2rg^tm1Wjl^*/SzJ (NSG) mice, the question came up that, even if a compound works to treat the cancer in these mice, would it translate the same for humans? As everyone has different genetics and responds to treatments differently, the main question that Jennifer and colleagues attempted to address in their winning publication was whether the genetic background of a mouse plays a role in the implantation and engraftment of different tumour types.

To answer this question, they generated or obtained diverse genetic strains that were lymphodeficient, and therefore could accept xenografts. They expected minimal difference in tumour growth between strains as the xenografted cell lines could grow on plastic. Unexpectedly, there was a significant difference between strains, suggesting that the genetics of the host microenvironment played a critical role in the development of cancer – that is how this manuscript was brought to life (Sargent et al., 2022).

Interestingly, Jennifer's previous work with mariculture and working with red-tide organisms was similar to what they observed in essence – the environment played a significant role in the quality of the shellfish harvested on the coast of Maine, implying that systems approaches to biology are essential in determining the mechanism of life. She looks forward to continuing to unravel the mysteries of systems biology in the future.

### Mark A. Warner

Mark's interest in science, mathematics and computers began at an early age, which led him to take advanced courses in these subjects at Mount Desert Island High School in Bar Harbor, Maine, USA. Mark's geometry teacher for his sophomore year of high school, Audrey Carter, saw that he was excelling at his work and encouraged him to assist other students who were struggling with concepts. In subsequent years, Mrs Carter was Mark's teacher for other mathematics courses and encouraged him to re-establish the Math Team. He recruited most of this team to represent the school in competitions against nearby schools. Mark also became a student aid for Mrs Carter's algebra class during his senior year.

Mark's interest in computers led him to attend the University of Maine at Farmington, pursuing a degree in computer science with a focus on programming for one year. Mark continued to take courses in mathematics and computer software at the University of Maine at Augusta through their continuing education program, while working in retail roles.

Mark knew that he wanted to pursue a career with a more fulfilling purpose, so he sought out a position at The Jackson Laboratory (JAX), where researchers study different diseases and make an impact on the world with their mouse models. As a first step towards achieving his goal of conducting scientific research, he accepted a position as an animal care trainee at the end of May 2017. At the end of his initial training period, he was selected to work in the Research Animal Facility (RAF) division, where animal care staff maintain research mice, to complete a secondary training period. Mark was selected for this division as he had expressed an interest in becoming a research assistant. In May 2018, Mark was promoted to Animal Care Technician I when an animal room became available for him to run in the RAF. By March 2019, another mouse room became available and Mark became a mid-level Animal Care Technician, running both mouse rooms. During his time working in animal care, Mark completed the Laboratory Animal Sciences and Genetics I and II courses that are taught at JAX.

In September 2020, Mark was hired as a Research Assistant I in the Patient-Derived Xenograft Research and Development Core (PDX R&D Core), a division of the Scientific Services at JAX. He quickly began to learn wet-laboratory techniques and surgeries used to xenograft mice using different methods. Mark also maintained the mouse colonies for the PDX R&D Core, working to produce several mouse strains that were *Rag1^−/−^* to allow for xenografting, some of which were used for the paper that won this award. In May 2022, Mark was promoted to Xenograft Studies Technician within the PDX R&D Core: he is currently continuing to learn more advanced xenograft techniques. During his time in the PDX R&D Core, Mark has also continued taking other courses offered through JAX, including courses in R and QuPath. He also plans to return to study computer science at college, focusing on data analysis and project management for STEM to further his career.

**DMM Prize 2022 shortlist**

**Hsp40 overexpression in pacemaker neurons delays circadian dysfunction in a *Drosophila* model of Huntington's disease.**

Pavitra Prakash, Arpit Kumar Pradhan and Vasu Sheeba. *Dis. Model. Mech.* (2022) 15, dmm049447. doi:10.1242/dmm.049447.

**Exercise suppresses mouse systemic AApoAII amyloidosis through enhancement of the p38 MAPK signaling pathway.**

Xiaoran Cui, Jinko Sawashita, Jian Dai, Chang Liu, Yuichi Igarashi, Masayuki Mori, Hiroki Miyahara and Keiichi Higuchi. *Dis. Model. Mech.* (2022) 15, dmm049327. doi:10.1242/dmm.049327.

**Vangl2–environment interaction causes severe neural tube defects, without abnormal neuroepithelial convergent extension.**

Oleksandr Nychyk, Gabriel L. Galea, Matteo Molè, Dawn Savery, Nicholas D. E. Greene, Philip Stanier and Andrew J. Copp. *Dis. Model. Mech.* (2022) 15, dmm049194. doi:10.1242/dmm.049194.

**Misregulation of Nucleoporins 98 and 96 leads to defects in protein synthesis that promote hallmarks of tumorigenesis.**

Ajai J. Pulianmackal, Kiriaki Kanakousaki, Kerry Flegel, Olga G. Grushko, Ella Gourley, Emily Rozich and Laura A. Buttitta. *Dis. Model. Mech.* (2022) 15, dmm049234. doi:10.1242/dmm.049234.

**Transcriptional targets of amyotrophic lateral sclerosis/frontotemporal dementia protein TDP-43 – meta-analysis and interactive graphical database.**

Maize C. Cao and Emma L. Scotter. *Dis. Model. Mech.* (2022) 15, dmm049418. doi:10.1242/dmm.049418.

**Limitations of mouse models for sickle cell disease conferred by their human globin transgene configurations.**

Kaitly J. Woodard, Phillip A. Doerfler, Kalin D. Mayberry, Akshay Sharma, Rachel Levine, Jonathan Yen, Virginia Valentine, Lance E. Palmer, Marc Valentine and Mitchell J. Weiss. *Dis. Model. Mech.* (2022) 15, dmm049463. doi:10.1242/dmm.049463.

**A genetic labeling system to study dendritic spine development in zebrafish models of neurodevelopmental disorders.**

Elisabeth C. DeMarco, George R. Stoner and Estuardo Robles. *Dis. Model. Mech.* (2022) 15, dmm049507. doi:10.1242/dmm.049507.

**Tissue architecture delineates field cancerization in BRAF^V600E^-induced tumor development.**

Elin Schoultz, Ellen Johansson, Carmen Moccia, Iva Jakubikova, Naveen Ravi, Shawn Liang, Therese Carlsson, Mikael Montelius, Konrad Patyra, Jukka Kero, Kajsa Paulsson, Henrik Fagman, Martin O. Bergo and Mikael Nilsson. *Dis. Model. Mech.* (2022) 15, dmm048887. doi:10.1242/dmm.048887.

**Molecular Subtyping Resource: a user-friendly tool for rapid biological discovery from transcriptional data.**

Baharak Ahmaderaghi, Raheleh Amirkhah, James Jackson, Tamsin R. M. Lannagan, Kathryn Gilroy, Sudhir B. Malla, Keara L. Redmond, Gerard Quinn, Simon S. McDade, ACRCelerate Consortium, Tim Maughan, Simon Leedham, Andrew S. D. Campbell, Owen J. Sansom, Mark Lawler and Philip D. Dunne. *Dis. Model. Mech.* (2022) 15, dmm049257. doi:10.1242/dmm.049257.

**Novel patient-derived models of desmoplastic small round cell tumor confirm a targetable dependency on ERBB signaling.**

Roger S. Smith, Igor Odintsov, Zebing Liu, Allan Jo-Weng Lui, Takuo Hayashi, Morana Vojnic, Yoshiyuki Suehara, Lukas Delasos, Marissa S. Mattar, Julija Hmeljak, Hillary A. Ramirez, Melissa Shaw, Gabrielle Bui, Alifiani B. Hartono, Eric Gladstone, Siddharth Kunte, Heather Magnan, Inna Khodos, Elisa De Stanchina, Michael P. La Quaglia, Jinjuan Yao, Marick Laé, Sean B. Lee, Lee Spraggon, Christine A. Pratilas, Marc Ladanyi and Romel Somwar. *Dis. Model. Mech.* (2022) 15, dmm047621. doi:10.1242/dmm.047621.

**A genetic screen in *Drosophila* reveals the role of fucosylation in host susceptibility to *Candida* infection.**

Marcus T. Glittenberg, Ilias Kounatidis, Magda Atilano and Petros Ligoxygakis. *Dis. Model. Mech.* (2022) 15, dmm049218. doi:10.1242/dmm.049218.

**Winner: Research Article.**

**Statins mediate anti- and pro-tumourigenic functions by remodelling the tumour microenvironment.**

Tamihiro Kamata, Esraa Al Dujaily, Salwa Alhamad, Tsz Y. So, Olga Margaritaki, Susan Giblett, J. Howard Pringle, John Le Quesne and Catrin Pritchard. *Dis. Model. Mech.* (2022) 15, dmm049148. doi:10.1242/dmm.049148.

**Winner: Resource Article.**

**Genetically diverse mouse platform to xenograft cancer cells.**

Jennifer K. Sargent, Mark A. Warner, Benjamin E. Low, William H. Schott, Todd Hoffert, David Coleman, Xing Yi Woo, Todd Sheridan, Sonia Erattupuzha, Philipp P. Henrich, Vivek M. Philip, Jeffrey H. Chuang, Michael V. Wiles and Muneer G. Hasham. *Dis. Model. Mech.* (2022) 15, dmm049457. doi:10.1242/dmm.049457.
